# Recent Advancements of Nanomedicine towards Antiangiogenic Therapy in Cancer

**DOI:** 10.3390/ijms21020455

**Published:** 2020-01-10

**Authors:** Anubhab Mukherjee, Vijay Sagar Madamsetty, Manash K. Paul, Sudip Mukherjee

**Affiliations:** 1 Aavishkar Oral Strips Pvt Ltd., 109/3, IDA, Phase 2, Sector 2, Lane 6, Cherlapally, Hyderabad 500051, India; anubhabrsv@gmail.com; 2 Department of Biochemistry and Molecular Biology, Mayo Clinic College of Medicine and Science, Jacksonville, FL 32224, USA; sagarchemistry@gmail.com; 3 Division of Pulmonary and Critical Care Medicine, David Geffen School of Medicine, The University of California, Los Angeles (UCLA), Factor Bldg. 10-240, 621 Charles E. Young Dr., Los Angeles, CA 90095, USA; 4 Department of Bioengineering, Rice University, Houston, TX 77030, USA

**Keywords:** angiogenesis, anti-angiogenesis, nanomedicine, cancer, theranostics

## Abstract

Angiogenesis is a process of generation of de-novo blood vessels from already existing vasculature. It has a crucial role in different physiological process including wound healing, embryonic development, and tumor growth. The methods by which therapeutic drugs inhibit tumor angiogenesis are termed as anti-angiogenesis cancer therapy. Developments of angiogenic inhibiting drugs have various limitations causing a barrier for successful treatment of cancer, where angiogenesis plays an important role. In this context, investigators developed novel strategies using nanotechnological approaches that have demonstrated inherent antiangiogenic properties or used for the delivery of antiangiogenic agents in a targeted manner. In this present article, we decisively highlight the recent developments of various nanoparticles (NPs) including liposomes, lipid NPs, protein NPs, polymer NPs, inorganic NPs, viral and bio-inspired NPs for potential application in antiangiogenic cancer therapy. Additionally, the clinical perspectives, challenges of nanomedicine, and future perspectives are briefly analyzed.

## 1. Introduction

Similar to normal tissues, tumors need nourishments by means of food and oxygen as well as a capacity to remove metabolic excretes and carbon dioxide. Diverse patterns of tumor-associated neovascularization, obtained by angiogenesis, cope with these demands. Angiogenesis, sprouting of new vessels from existing quiescent ones, remains almost always turned on during the process of tumorigenesis for sustenance of neoplastic expansions [1,2,3]. A riveting account of studies corroborates that the “angiogenic switch” is regulated by counterbalancing factors like signaling proteins for induction (vascular endothelial growth factor (VEGF)-signaling via three receptor tyrosine kinases, etc.) or inhibition (thrombospondin-1, etc.) of angiogenesis and gets inclined towards angiogenesis when stimulated by hypoxia or inflammation [4,5,6,7,8,9,10,11,12]. Other pro-angiogenic signals fibroblast growth factor ((FGF) family members, transforming growth factor-β, etc.), when persistently upregulated, also contribute to sustain tumor angiogenesis [5,13]. Amid all plausible ways to treat cancer, anti-angiogenic therapy—targeting tumor vasculature to prevent aberrant capillary sprouting—has received an astounding outpouring of research in the last few decades [14,15].

The idea to decouple tumors from surrounding blood vessels has led to discovery and clinical approval of several anti-angiogenic drugs, namely, monoclonal antibody inhibitors (Bevacizumab, IMC-1121B, 2C3), receptor tyrosine kinase inhibitors (sorafenib, sunitinib, pazopanib), soluble receptor chimeric protein (VEGF-Trap), inhibitors of endothelial cell proliferation (thalidomide, angiostatin), inhibitors of integrin’s proangiogenic activity (Cilengitide, medi-522), matrix metalloproteinase inhibitors (Neovastat, Prinomastat, Marimastat), vascular targeting drug (combretastatin), etc. [16,17,18,19]. However, many of these meteoric developments, alone or in combination with chemotherapy or radiotherapy, were vitiated by sparse clinical efficacy to combat tissue invasion and metastases. Drug resistance, upregulation of various proangiogenic signals, hypoxia resistance, delayed response to radiotherapy, toxicity issues, etc., have hindered the preponderance of antiangiogenic therapy [8,20,21,22,23].

To this end, nanotechnology offers an attractive biomedical platform, involving smart design of vehicles with unique physicochemical properties for targeted delivery and sustained release of therapeutics at the site of action along with their tracking details, which holds the promise to circumvent the existing limitations [13,22,24,25,26,27]. In the present review, we shall elaborately discuss the limitations of current therapies, advent of nanomedicine as an alternative modality in antiangiogenic cancer therapy including lipid-based and polymer-based nanoparticles, inorganic nanoparticles, protein and viral based nanocarriers, their safety, challenges, clinical outlook, and future perspectives.

## 2. Cancer, Statistics, Conventional Therapy, Challenges

Cancer is the leading cause of death throughout the world and is second to cardiovascular diseases and causes enormous health and economic burden. As per the World Health Organization (WHO) and an approximate of 9.6 million people died worldwide due to cancer in 2018 only. In 2019 alone, an estimated 1,762,450 new cancer cases were reported and approximately 606,880 cancer deaths happened in the United States. The data presented by the National Center for Health Statistics (NCHS) is promising as it shows an overall decrease in cancer date rate by 27% during the period of 1991 to 2016. Conventional cancer therapies have several limitations and associated side effects. The data presented by NCHS, WHO, and NCI suggests that early detection, and diagnosis coupled with better treatment strategies can reduce the burden of cancer [28,29]. Angiogenesis promotes tumor progression and metastasis. Metastatic disease is overwhelmingly the predominant cause of cancer death [30]. Several data suggest that angiogenesis is the prerequisite for the dissemination and establishment of metastatic tumor cells to distant organs. Hence combination therapies with emphasis on anti-angiogenic and antilymphangiogenic treatments to prevent the spread of cancer [31]. Understanding the morphology and molecular differences of the newly formed angiogenic blood vessels is critical in designing antiangiogenic therapies. Considering the success of antiangiogenic treatment and advances made in nanoparticles-based specific delivery of therapeutics to the newly formed blood vessels during neo-angiogenesis and tumor infiltration may reduce the cancer burden significantly. Considering, the recent success in immunotherapy and cancer vaccination and the close link between immune microenvironment and angiogenesis is unique. Combinations should be used to reduce the cancer burden [21]. Nanobiotechnology may play a key role in effective detection, targeting, and delivery of immune-therapeutics and antiangiogenic therapeutics at the tumor site. Nanotechnology and nanoparticles offer several advantages like increased half-life, reduced toxicity, specific and selective delivery over the free drugs/therapeutics [13,22,26]. Nanovectors have also opened up new avenues for noninvasive imaging and can be successfully used to create angiogenic maps for patient specific therapeutic planning [21].

## 3. Angiogenesis and Cancer

Angiogenesis is the process of formation of new blood vessels orchestrated by proangiogenic and antiangiogenic factors during development, reproduction, and repair. Of utmost importance is pathological angiogenesis, especially in the context of neoplastic disease. Tumor cells rapidly proliferate and need a continuous supply of oxygen and nutrients and thereby requires steady requirement for infiltrating blood vessels. Angiogenesis is one of the hallmarks of cancer [32]. Targeting the angiogenic process is regarded as a logical approach to the treatment of various malignancies and various antiangiogenic treatment agents have been developed and tested in clinical trials. The concept of antiangiogenic therapy came into being while Dr. Folkman discovered that tumors require steady oxygen and nutrient supply, and the neogenesis of tumor-infiltrating vessels helps sustain the continuous growth of the tumor. The inhibition of angiogenesis can lead to tumor starvation and tumor cell death [33,34]. Angiogenesis or the formation of new blood vessels from pre-existing vasculature is the common mechanism of new angiogenesis [35]. Angiogenic blood vessel formation may occur by three major processes, sprouting angiogenesis, vasculogenesis, and intussusception. Sprouting angiogenesis is considered the most critical process of blood vessel formation in the tumor but evidence suggests that a non-angiogenic process called vessel co-option is also essential. Tumor cells opt for vessel co-option at different anatomical sites by hijacking pre-existing blood vessels from the normal neighboring issue [36,37].

Hypoxia is the key factor that regulates tumor angiogenesis. Tumor cells grow rapidly and are hypoxic in nature, and secrete vascular endothelial growth factor A (VEGFA). Not only the tumor cells but also the tumor associated stromal cells produce proangiogenic factors. The endothelial cells in the microenvironment express VEGF receptor (VEGFR2). The gradient of VEGF can be sensed by the endothelial cell based on the VEGF–VEGFR interaction; this leads to the formation of new endothelial sprout towards the tumor cell [35,38].

Tumor cell induces a complex cascade of angiogenic signaling and activates downstream cellular events in multiple cell types, especially the endothelial cells, leading to angiogenesis. Proangiogenic factors include fibroblast growth factor (FGF) families, vascular endothelial growth factor (VEGF), platelet-derived growth factor (PDGF), transforming growth factors-alpha/beta (TGF-α/β), and angiopoietin (Ang 1,2) and the associated receptors. The detachment of the perivascular cells from the mature blood vessels initiates vessel remodeling and endothelial cell proliferation. Platelets become activated and recruited to the sites of the exposed basement membrane. Tumor-associated macrophages (TAM) produce angiogenic factors such as VEGF, Matrix metalloproteinases (MMPs), and urokinase-type plasminogen activator (uPA). On the other hand, precursor endothelial cells move to the perceived wound site and release angiogenic factors. The activated endothelial cells release proteases and lead to extracellular matrix (ECM) remodeling, followed by directional sprouting. The signaling cascades activate tube formation and branching, followed by vessel arterio-venous patterning and maturation. Cancer progenitor/stem cells can differentiate to endothelial cells, and thereby participates directly in angiogenesis.

**Figure 1:**
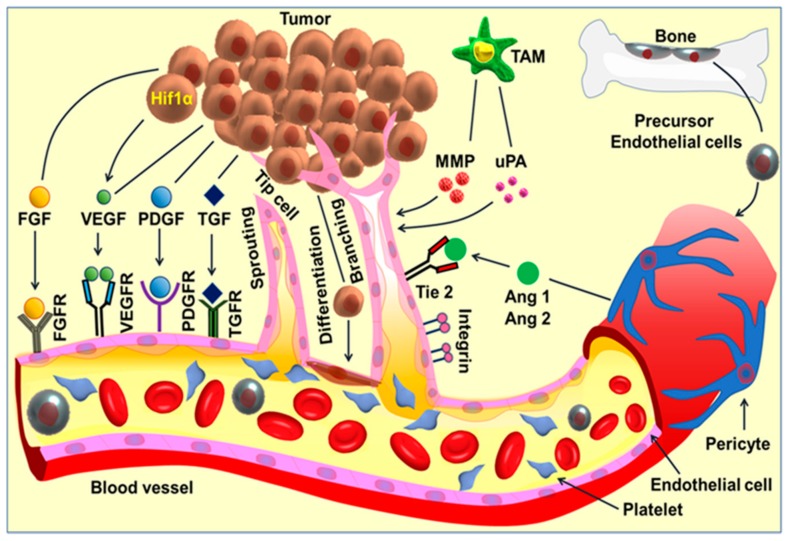
Angiogenic signaling pathway and angiogenesis. Tumor cell induces a complex cascade of angiogenic signaling and activates downstream cellular events in multiple cell types, especially the endothelial cells, leading to angiogenesis. Pro-angiogenic factors include fibroblast growth factor (FGF) families, vascular endothelial growth factor (VEGF), platelet-derived growth factor (PDGF), transforming growth factors-alpha/beta (TGF-α/β), and angiopoietin (Ang 1,2) and the associated receptors. The detachment of the perivascular cells from the mature blood vessels initiates vessel remodeling and endothelial cell proliferation. Platelets become activated and recruited to the sites of the exposed basement membrane. Tumor-associated macrophages (TAM) produce angiogenic factors such as VEGF, MMPs and urokinase-type plasminogen activator (uPA). On the other hand, precursor endothelial cells move to the perceived wound site and release angiogenic factors. The activated endothelial cells release proteases and lead to extracellular matrix (ECM) remodeling, followed by directional sprouting. The signaling cascades activate tube formation and branching, followed by vessel arterio-venous patterning and maturation. Cancer progenitor/stem cells can differentiate to endothelial cells, and thereby participates directly in angiogenesis.

The process of vessels sprouting is a coordinated process including the tip/stalk cell selection, followed by tip cell directed migration, stalk cell proliferation, branching coordination, elongation of stalk, lumen formation, and vessel maturation [39]. Additional signaling molecules like delta-ligand-like 4 (DLL4) and angiopoietin 2 (ANGPT2) also play an important role in the process of angiogenesis. Until proangiogenic signals fade away, the establishment of the basement membrane, the sprouting and branching continues on, followed by vessel maturation. In cancer, however, the angiogenesis process is rapid but considerable variation exists in the vessel morphology, functionality, and integrity [40]. Figure 1 is an overall presentation of angiogenic signaling pathway and angiogenesis.

## 4. Current Antiangiogenic Therapies in Cancer and Their Limitations

Angiogenesis inhibitors are designed to target existing tumor infiltrating blood vessels and inhibit the formation of newly formed blood vessels and thereby halt the tumor metabolism and growth. Based on the inherent nature of the neo-angiogenesis in primary tumors, angiogenesis inhibitors might activate the destruction of immature angiogenic vessels or stop the angiogenic switch thereby preventing vascular metastasis [13]. Angiogenic inhibitors in conjunction with combination chemotherapy, may increase the antitumor potency to a significant level as these agents can halt the tumor progression but not eliminate them. There are two types of angiogenesis inhibitors; the first group includes direct inhibitors, agents that block main angiogenesis proteins. The second group comprises indirect inhibitors that target the tumor cell or stromal cells and modulate angiogenesis regulators [41]. The direct angiogenesis inhibitors exert their antiangiogenic effect by binding with angiogenesis inducers like VEGF, bFGF, and PDGF. Bevacizumab was the first antiangiogenic therapy that was approved by the FDA in 2004 for the treatment of colorectal cancer. Antiangiogenic treatment is the fourth modality of cancer therapy, as the treatment targets microvascular endothelial cells in the tumor microenvironment and not the tumor directly [13,42]. Fast-growing tumors need greater microvasculature and are more susceptible to antiangiogenic therapy. Table 1 shows the balance between pro and antiangiogenic factors is the key to antiangiogenic treatment. Table 1 shows the chemical structure and the mechanism of action of a list of FDA approved angiogenesis inhibitors for different cancers. Several clinical trials are in progress that can induce modest disease-free survival advantages. Table 1 also shows a list of potential drugs that have demonstrated antiangiogenic properties in preclinical trials.

Although the concept of targeting tumor angiogenesis has opened up new possibilities to treat cancer, many limitations need to be addressed to make this therapy successful. Toxicity is one of the main drawbacks of antiangiogenic treatment, the main side effects being hemorrhage, hypertension proteinuria, thrombosis, and poor wound healing. Preclinical data have suggested that the medication affects not only the tumor vasculature but also the vasculature in multiple organs, especially vasculature rich organs. Optimal dose calculation, especially in combination chemotherapy, is very challenging. Current decisions in chemotherapy management do not consider inter-tumoral vessel vasculature to decide the dose and type of antiangiogenic therapy [60,61]. Another drawback is the development of acquired resistance to antiangiogenic therapy, causing transient disease stabilization and activation of alternative pathways, vessel co-option, vessel mimicry, and enhanced metastasis. Cancer cells can develop resistance to antiangiogenic treatment by multiple mechanisms [61]. Cancer cells upregulate proangiogenic factors like angiopoietins (Ang), epidermal growth factor (EGF), fibroblast growth factor (FGF), interleukin 8 (IL-8) etc., to activate compensatory pathways to stimulate blood vessel formation [60,61]. Activation of hypoxia inducing factor (Hif1) and downstream ZEB2 are the alternative angiogenic pathway that can be targeted.

Cancer cells also secrete several growth factors that recruit cells that ultimately cause resistance to therapy. Bone marrow-derived cells, monocytes, macrophages, endothelial precursor cells, myeloid-derived suppressor cells, and cancer-associated fibroblasts can infiltrate and produce proangiogenic factors which might lead to VEGF-independent new blood vessel formation. The association between the immune system and angiogenesis is not well understood and is a bottleneck in antiangiogenic therapy. Vascular heterogeneity, especially in tumor vessels, is quite remarkable, leading to a variable degree of pericyte coverage and VEGF expression; vessel leakage can thereby lead to altered anti-VEGF response [62,63]. Non-angiogenic cancers exploit the process of vessel co-option (by hijacking pre-existing vessels) and thereby cause resistance to antiangiogenic therapy as evident from the results of several clinical trials [63]. The other potential mechanism operations can be extracellular vesicles, post-translational modification, genetic polymorphism. Therefore, the knowledge of acquired resistance will help design theranostic drugs.

## 5. Alternative Therapy: Nanomedicine

**Figure 2:**
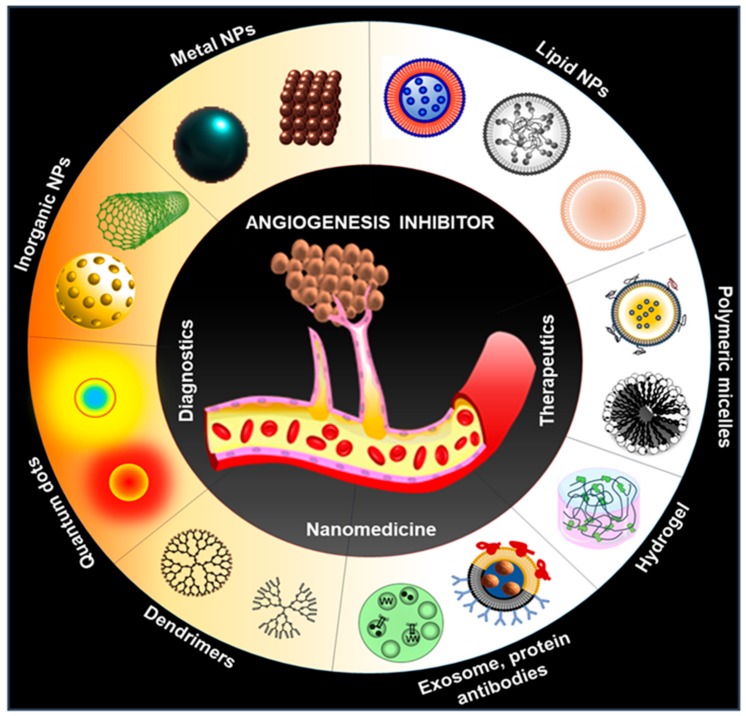
Schematic representation of multiple nanomedicine approaches that may be used for the diagnosis and treatment of angiogenesis.

In this context, nanomedicine plays an important role to overcome the existing limitations of present antiangiogenic therapy due to their interesting physicochemical properties (small size and high surface area) at nanoscale. Recently, many investigators as well as our group exhibited various, multifunctional theranostics applications of nanomedicine in different diseases including cancer, diabetes, neurodegenerative disease, cardiovascular diseases, antibacterial, spinal cord injury, etc. [23,25,64,65,66]. Nanoparticles conjugated to various targeting ligands can be employed to utilize active targeting of antiangiogenic drugs for better therapeutic efficacy. Additionally, various reports showed the anti and proangiogenic properties of several inorganic nanoparticles (NPs) including silver NPs (AgNPs), gold NPs (AuNPs), copper nanoparticles (CuNPs), carbon nanotubes (CNT), europium hydroxide nanorods (EHNs), graphene oxides (GO), zinc oxide nanoflowers, and cerium oxide nanoparticles (NCe) [13,22,67,68]. Moreover, various other nanomaterials including liposomes, lipid NPs, protein NPs, polymer NPs, viral and bio-inspires NPs are utilized for the targeted delivery of antiangiogenic agents to tumors for the suppression of tumor angiogenesis [26,69,70,71,72] (Figure 2). Active targeting of antiangiogenic drugs also help to reduce the unwanted side effects and toxicity.

## 6. Lipid-Based Nanoparticles for Antiangiogenic Therapy

**Figure 3:**
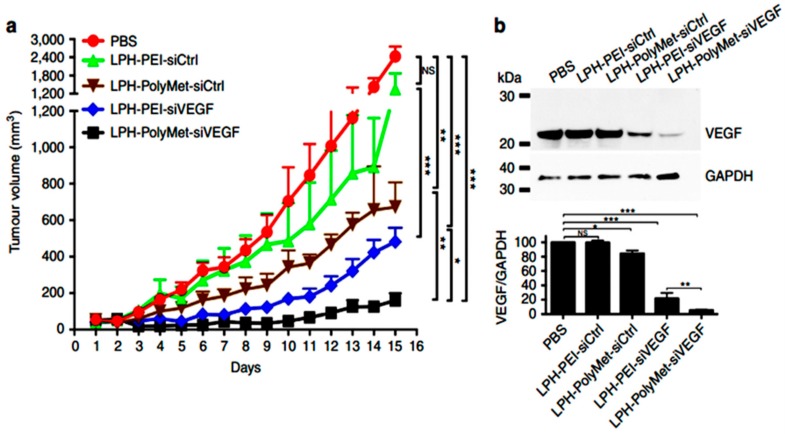
Lipid nanoparticles made up of PolyMet can systemically deliver vascular endothelial growth factor (VEGF)siRNA to the tumor site and inhibit tumor growth. (**a**) H460 tumor-bearing mice were injected i.v. every other day and tumor volumes were measured every day. (**b**) H460 tumor VEGF protein levels after two injections were measured by Western blot analysis. The bar chart in (b) represents the quantitative analysis of relative normalized VEGF band intensity (Image J). Data are mean ± s.e.m. (n ¼ 5 per group) analyzed by two-way analysis of variance with Tukey’s correction. Data are representative of (b) or combined from (a) three independent experiments. NS, not significant; * *p* < 0.05, ** *p* < 0.01, *** *p* < 0.005. Reproduced with permission from [74]. Copyright, 2016, NPG.

Amid a variety of nanocarriers, lipid-based systems such as liposomes, solid lipid NPs, lipid-polymer hybrid NPs, and nanostructured lipid carriers are most widely explored for cancer therapeutics including antiangiogenic therapeutics, as lipid-based vehicles have advantages to solubilize insoluble drugs, encapsulate multiple hydrophobic and hydrophilic drugs, and deliver them at specific tissue sites to accomplish enhanced bioavailability while avoiding off-target side-effects. In this section, we will highlight the recent advancements in lipid-based NPs in antiangiogenic therapy. As discussed earlier, VEGF and its receptors (VEGFRs) play a crucial role in angiogenesis and proliferation of many type of cancer cells, including melanoma, breast, lung, and brain cancer [73]. Downregulation of VEGF expression or inhibiting its receptors activity through various methods, thus, was proposed to efficiently suppress tumor angiogenesis with simultaneous tumor growth inhibition (in combination with some other chemotherapeutics). Recently, Prof. Leaf Huang’s laboratory developed a polymetformin (PolyMet) containing hyaluronic acid decorated lipid NPs for systemic gene delivery. PolyMet NPs were shown to be highly capable of VEGF siRNA delivery for VEGF knockdown in a human lung cancer xenograft, leading to enhanced tumor activity by inhibiting angiogenesis [74] (Figure 3). The same group also developed lipid-based dual functionalized NPs to VEGF siRNA in vivo [75]. Recently, Yang and his colleagues developed a low-density lipoprotein receptor-related protein receptor (Angiopep-2) and neuropilin-1 receptor (tLyP-1) targeting cationic liposomes for delivery of siRNA and docetaxel to gliomas. The dual peptide-decorated liposomes showed efficacious antiangiogenic activity by knocking down VEGF siRNA along with antiproliferative apoptotic effects exerted by docetaxel in U87 MG tumors [76]. Dr. Wang’s laboratory developed a miRNA loaded cRGD-functionalized lipid NPs for antiangiogenic therapy [77]. Another report showed promising VEGF inhibition results by using dual receptor targeting liposomes [78]. Guo and his team demonstrated that Lcn2 siRNA loaded ICAM-1-targeting liposomes showed a potent antiangiogenic effect in triple-negative breast cancer (TNBC) [79]. Several other research groups also developed similar lipid-based NPs for gene-mediated angiogenesis inhibition [80,81,82,83,84,85,86,87]. Sorafenib inhibits angiogenesis and proliferation via binding to VEGFR-2, VEGFR-3, and PDGFR-b tyrosine kinases. The clinical trials of sorafenib are suggesting that it displays a high antiangiogenic efficacy in several cancers and it also strengthens the efficacy of other chemotherapeutics. However, very poor water solubility and off-target side effects of sorafenib limit its clinical usage. Scientists are using nanotechnology tools to overcome these issues. For example, Meneiand his team developed sorafenib-encapsulated lipid NPs for the treatment of glioblastoma, which showed high efficiency in the suppression of angiogenesis by inhibiting CD31 [88]. More recently, Zang and his group demonstrated that co-delivery of VEGF-siRNA and Sorafenib through pH-sensitive liposomes showed a synergistic effect in hepatocellular carcinoma [89]. Several other lipid-based NPs, along with other chemotherapeutics, are also being used for better antiangiogenic and antitumor therapy [90,91]. mTOR inhibitor like rapamycin and its analogs also proved their antiangiogenic capability. Many studies confirmed that these analogs inhibit the expression of VEGF in tumor cells. Recently, several studies elicited that rapamycin and its analogs, loaded as single and in combination with other drugs onto lipid-based NPs, could be used for effective antiangiogenic therapy [92,93,94,95].

There is a piece of evidence that somatostatin receptors (SSTRs), mainly subtype 2 (SSTR2), are significantly expressed in both glioma and glioma vasculature endothelial cells. Recently, Misra’s lab developed paclitaxel (PTX) loaded solid lipid NPs (SLN) functionalized with Tyr-3-octreotide (ligand for SSTR2) to facilitate dual-targeted chemotherapy by targeting both brain tumor and tumor neovasculature cells. The study demonstrated excellent tumor growth inhibition and enhanced survival by an antiangiogenic (CD31 inhibition) and antitumor effect of PTX in orthotopic glioma-bearing rats. Additionally, the authors studied tumor vasculature and tumor targeting efficiency of NPs by conjugating^99^ mTc [96].In another recent study, the authors demonstrated significant suppression of angiogenesis by targeting oxaliplatin loaded PEGylated cationic liposomes in a dorsal air sac mouse model [97]. Earlier this century, Sengupta et al. [98] and Ebos et al. [20] developed polymer lipid hybrid nanocarriers for delivery of combretastatin (an anti-angiogenesis drug) along with doxorubicin as a chemotherapeutic. In summary, there is an enormous amount of progress observed in lipid-based antiangiogenics.

## 7. Polymeric Nanomedicine

Among all the commonly used biodegradable materials, polymers offer a superior advantage in the drug delivery field for tumor angiogenesis. Poly (lactic-co-glycolic acid) (PLGA) is a widely used, FDA approved biocompatible polymer, which offers a versatile platform to load multiple hydrophobic and hydrophilic small molecule drugs or in combination using various emulsion procedures [99,100].

After Judah Folkman unequivocally enunciated the “angiogenic switch” hypothesis for tumor progression in 1991, angiogenesis has become an important component of tumor growth and development and there has been an incredible rush in targeting angiogenesis for cancer therapeutics [101]. Therefore, there is an urgent need for efficient angiogenesis inhibitors development. O-(chloracetyl-carbamoyl) fumagillol (TNP-470, angiogenesis inhibitor) reduced tumor growth in patients with metastatic cancer. However, at required higher doses, many patients experienced neurotoxicity. To overcome this, Folkman and his team developed a water-soluble TNP-470 conjugated 2-Hydroxypropyl methacrylamide (HPMA) copolymer and nanopolymeric micelles (Lodamin). These formulations demonstrated beneficial drug delivery features, such as prolonged systemic circulation half-life, targeting capabilities, controlled drug release, and used as oral nontoxic antiangiogenic drugs [102,103].

**Figure 4:**
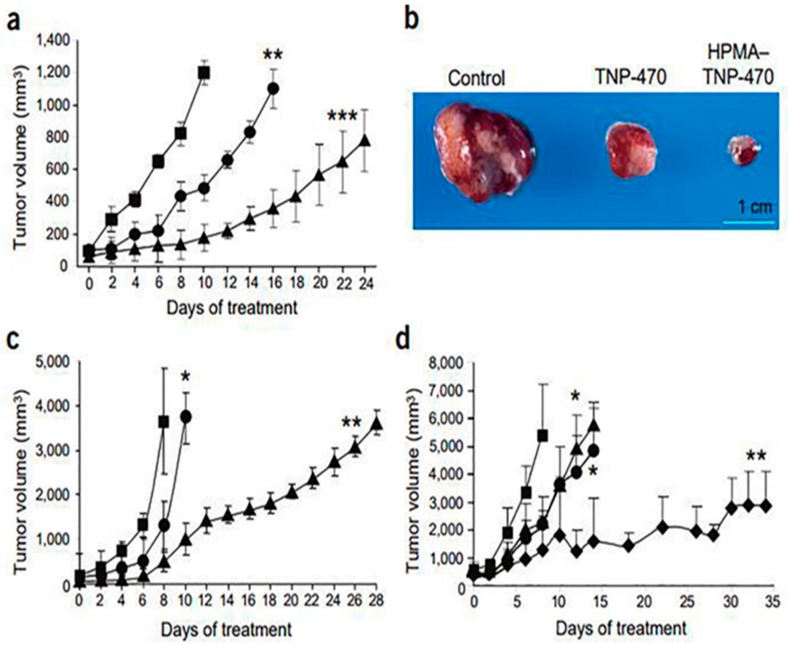
HPMA copolymerTNP-470 inhibitsA2058 human melanoma and LLC growth. (**a**) Effects of TNP-470 (●), HPMA copolymerTNP-470 conjugate (▲) and saline (█) on male SCID mice bearing A2058 human melanoma (*n* = 5 mice per group). (**b**) Excised tumors (from (a)) on day 8 of treatment. (**c**) Effects of TNP-470 (30 mg/kg q.o.d. s.c.; ●) and HPMA copolymerTNP-470 (30 mg/kg q.o.d. s.c.; ▲) on C57 mice bearing LLC tumors and untreated control mice (█); *n* = 10 mice per group). (**d**) Dose escalation of HPMA copolymerTNP-470 inC57 mice bearing LLC tumors. Data at 30 (▲), 60 (●), and 90 mg/kg q.o.d. (♦) and controls (█) are shown (*n* = 5 mice per group). All data represent mean ± s.e. * *p* < 0.05; ** *p* < 0.03; *** *p* < 0.01 compared with control [102]. Reproduced with permission from [102]. Copyright, 2004, NPG.

Importantly, as shown in Figure 4, TNP-470 conjugated HPMA copolymer significantly inhibitedA2058 human melanoma and Lewis lung carcinoma (LLC) tumor growth which suggesting compelling future antiangiogenic and anticancer treatment options for patients [102]. In another study, Harfouche et al. described LY294002 loaded PLGA nanoparticles, which can efficiently inhibit melanoma tumor growth by inducing apoptosis in zebrafish tumors [104]. A combination of chemo- and anti-angiogenesis therapy holds immense potential for effective tumor growth inhibition. For example, Yao and his group developed heparin–gambogic acid-containing and c(RGDyK)-functionalized self-assembled polymeric amphiphilic nanosystem. This formulation showed considerable inhibition of VEGF, hypoxia inducible factor-1 alpha, and CD31 expression with significant downregulation of pVEGFR2. These results offer a versatile nanoplatform for efficient combinatorial tumor therapy [105]. In a similar study, nanopolymer was developed for targeted co-delivery of multiple anticancer and antiangiogenic agents using LyP-1 peptide as a targeting ligand [106]. Later on, several other hybrid polymers have been developed for antiangiogenic therapy; for example, mitomycin C and doxorubicin co-encapsulated polymeric.

**Figure 5:**
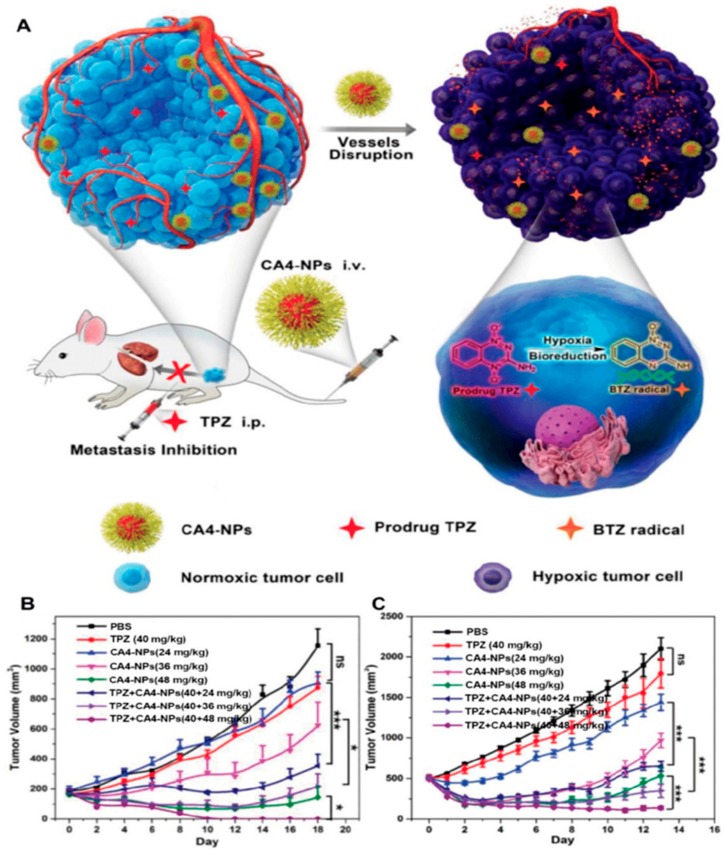
Schematic illustration of hypoxia-inducing vascular disrupting agents (VDA) nanodrug combined with hypoxia-activated prodrug for cancer therapy (**A**). Tumor volume changes of BALB/c mice bearing 4T1 tumors with both moderate sizes (≈180 mm^3^) (*n* = 6) (**B**) and large sizes (≈500 mm^3^) (*n* = 6). (**C**). All data points are presented as mean ± standard deviation (s.d.) (* *p* < 0.05, ** *p* < 0.01, *** *p* < 0.001). Reproduced with permission from [109]. Copyright, 2019, Wiley-VCH.

Nanoparticles exhibited superior anti-angiogenesis and antitumor activity with minimal systemic toxicity in both sensitive and drug-resistant orthotopic xenografts of breast cancer [107]. Lung metastasis is one of the primary causes of mortality with no cure available currently. The dual-treatment options, such as, targeting anticancer and anti-angiogenesis agents may offer some advantages. Recently the same group developed a similar approach using RGD peptide as a targeting moiety and demonstrated significant inhibition of the lung metastasis progression and extended median survival [108]. As shown in Figure 5, Chen and coworkers developed a poly(L-glutamic acid)-CA4 containing polymeric NPs for selective disruption of unusual tumor vasculature, in addition to elevating the hypoxia level of the tumor microenvironment to further boost up the antitumor ability of Tirapazamine in metastatic tumors [109]. Additionally, developments in gene therapy for antiangiogenic cancer therapy has become more attractive [110]. For example, therapeutic gene combinations of siMyc, siVEGF, and siBcl-2 and an imaging agent containing poly(d,l-lactide-co-glycolide) (PLG) multi-model nanomaterials functionalized with rabies virus glycoprotein (RVG) peptide for neuroblastoma-targeting delivery showed potential antitumor efficacy in a neuroblastoma mouse model [111]. A similar study was performed using block catiomer of poly (ethylene glycol) (PEG)-b-poly[*N*′-[*N*-(2-[aminoethyl)-2-aminoehtyl]aspartamide]-cholesteryl conjugated with RGD and an antiangiogenic gene which demonstrated promising results [112]. For interested readers, we provide a few more reviews describing recent advances of nanotherapeutic-based cancer starvation therapy, challenges, and future prospects of these anticancer strategies [21,71,113,114,115].

## 8. Inorganic Nanoparticles

Inorganic nanoparticles including AuNPs, AgNPs, CNTs, PtNPs, CuNPs, ZnO, and Fe_3_O_4_, etc., have gained immense attention due to their multifunctional properties, easy synthesis, easy functionalization, and inherent pro and/or antiangiogenic properties [22,25,26]. Several investigations, including ours, demonstrated the antiangiogenic properties of various inorganic nanomaterials in cancer therapy in recent times [13,22,116,117].

**Figure 6:**
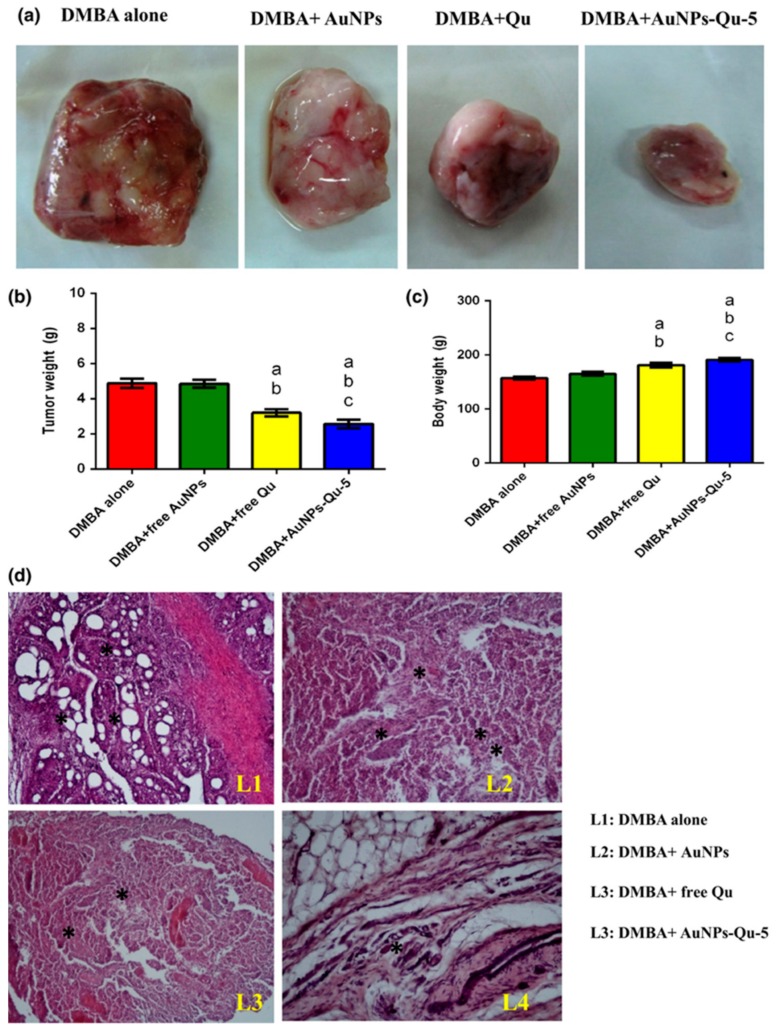
Effect of free Qu and AuNPs-Qu-5 on DMBA-induced mammary carcinoma in Sprague-Dawley rats. AuNPs-Qu-5 inhibited the DMBA-induced tumor growth in Sprague-Dawley rats: (**a**) representative photographs of breast tumors in each group; (**b**) weight of breast tumors in each group; (**c**) body weight of all the animals in each group. “a” DMBA alone vs. others; “b” DMBA induced animals +free AuNPs vs. others; and “c” DMBA induced animals + free Qu.(**d**) Effect of free Qu and AuNPs-Qu-5 on histopathological examination. (**d**) Histopathological examination of DMBA-induced breast cancer in Sprague-Dawley rats. Histopathological changes in the mammary tissues of cancer-induced vehicle and experimental animals (hematoxylin and eosin, 10×). L1: Cancer-induced breast cancer animals show the extensive solid areas and several neoplastic cells lobular structural disruption; L2: CI + free AuNPs show extensive solid tumors; L3: free quercetin-treated animal shows a small amount of neoplastic structure; L4: AuNPs-Qu-5-treated animal shows normal mammary epithelial cells appearance.*Represents neoplastic cells.

Mukherjee et al. demonstrated that 5 nm sized AuNPs inhibited the function of VEGF165 (HBGF) demonstrating anti-angiogenesis [67]. Balakrishnan and co-workers reported the use of chemically synthesized gold nanoparticle conjugated with naturally available photochemical quercetin for the inhibition of tumor angiogenesis, epithelial–mesenchymal transition, and tumor metastasis via EGFR/VEGFR-2-controlled pathway in in vitro and in vivo breast cancer (Figure 6) [118]. Pan et al. showed the antigenesis and tumor inhibition of AuNPs by inhibiting VEGF165-influenced VEGFR2 and phosphorylation of AKT pathways in mouse xenograft models [119]. Gurunathan et al. exhibited the antiangiogenic properties of biosynthesized silver nanoparticles using *Bacillus licheniformis* [120] observed by several in vitro (cell proliferation, tube formation, migration etc.) and in vivo (Matrigel plug assay) models. Song et al. showed the inhibition of angiogenesis using CuNPs causing inhibition of HUVEC migration, tube formation, and cell cycle arrest at various doses of treatment [121]. Giri et al. reported the use of nanoceria (NCe) nanoparticles for the inhibition of ovarian cancer growth in an in vivo mouse model that caused activation of MMP2 apart from inhibition of endothelial cell proliferation and migration [122]. In another recent report, Setyawati et al. demonstrated the antiangiogenic and antitumor activities of mesoporous silica nanoparticles in a size-dependent manner causing generation of ROS further activating the tumor suppressing p53 signaling pathways [123].

Mukherjee et al. showed the dose-dependent manipulation of anti-angiogenesis and angiogenesis using the treatment of graphene oxides by the modulation of ROS in in vitro (endothelial cell proliferation assay, scratching assay, and tube formation assay) and ex vivo models (chicken embryonic angiogenesis assay) [72].

## 9. Protein Based Nanoparticles

**Figure 7:**
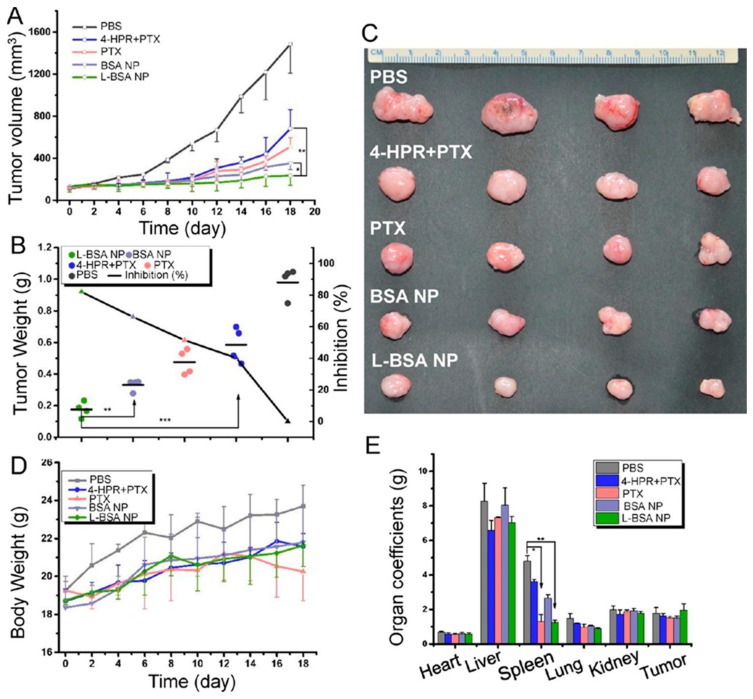
Antiglioma efficacy on the subcutaneous glioma mouse model. (**A**) Tumor growth curve. (**B**) Tumor weight and tumor inhibition rate. (**C**) Representative tumor tissues. (**D**) Bodyweight variations in the treatment course. (**E**) Organ coefficients (* *p* < 0.05, ** *p* < 0.01). Reproduced with permission from [128]. Copyright, 2016, ACS.

Protein-based NPs have also attracted substantial attention owing to their high biodegradability, highly symmetrically organized edifices, ideal size for delivery, ability for different interfaces functionalization, etc. [124,125,126,127]. For example, as shown in Figure 7, Lin et al. developed albumin based NP encapsulating paclitaxel and 4-HPR (angioprevention vitamin A analog) functionalized with blood brain barrier (BBB) crossing targeting peptide. These dual drug-loaded NPs showed excellent anti-glioma efficacy on the subcutaneous glioma mouse model by inhibiting angiogenesis, regulating tumor immune microenvironment, andinducing apoptosis [128]. Itraconazole (ITA) is originally an antifungal drug, but in recent years, it is being used in cancer as a multitarget anti-angiogenesis agent [129]. ITA inhibits several angiogenic pathways, including VEGF, VEGFR-2, FGF, etc. [129]. However, its poor water solubility is obstructing its usage as an effective antiangiogenic drug. Recently, Zhang and his team developed an ITA encapsulated BSA-NP for effective antiangiogenic and antitumor therapy on a patient-derived xenograft NSCLC model [129]. Rapamycin is one of the best angiogenic inhibitors that blocks angiogenesis by inhibiting downstream signals such as mTOR. Nevertheless, its reduced chemical stability, poor water solubility, and significant side effects limit its usage. Desai and his team employed albumin-based NP to overcome all issues related to rapamycin and demonstrated superior antiangiogenic and antitumor efficacy using nab-rapamycin in combination with nab-paclitaxel in human colon and breast cancer xenograft models. In another study, researchers developed PEG-modified gelatin-based nanovectors for sFlt-1 plasmid DNA delivery to solid tumor xenograft of breast cancer [130]. In summary, protein-based NPs provide a large contribution to antiangiogenic cancer therapy.

## 10. Viral and Other Bio-Inspired Nanoparticles

**Figure 8:**
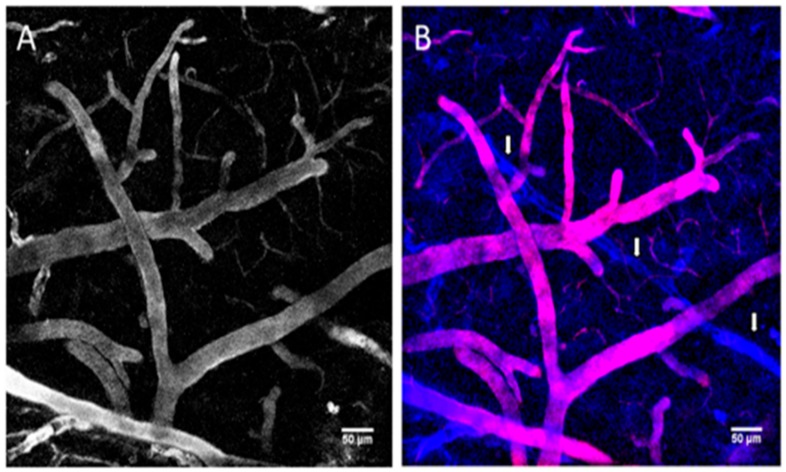
Intravital imaging of the mouse brain vasculature with Tobacco mosaic virus (TMV)-BF3 particles. (**A**) Mouse brain vessels labeled with TMV-BF3 at 1 h after intravenous injection into the tail vein. (**B**) Same observation window as shown in (A) but after a second injection, this time with sulforhodamine B; blue, fluorescence emitted from TMV-BF3; red, fluorescence emitted from sulforhodamine B. The 3D projections were performed with Fiji software using the standard deviation projection method. Reproduced with permission from [140]. Copyright, 2016, Frontiers.

During prior decades, the understanding of viral-based nanotechnology has improved, simultaneous with development in their design and production [131,132]. Among various virus-based nanoparticles (VNPs), plant-derived VNPs are considered safe from a human health perspective because they are not pathogenic in mammals and proteinaceous plant VNPs have appeared as a key platform for engineering with multiple drugs, imaging molecules, and targeting ligands [133,134]. VNPs emanated in various sizes, shapes, and each virus species is highly symmetrical and monodisperse. Numerous VNPs including plant viruses including Cowpea mosaic virus (CPMV), the Human papillomavirus (HPV), Brome mosaic virus (BMV), Cowpea chlorotic mottle virus (CCMV), Red clover necrotic mottle virus (RCNMV) and Hibiscus chlorotic ringspot virus (HCSRV) are materialized for various nanomedical applications [134,135]. Recently, VNPs are also being used in antiangiogenic therapy. For example, in 2019, Gamper et al. developed a nanocarrier containing coat protein (CP) of Tobacco mosaic virus (TMV) fused with a highly hydrophobic, insoluble peptide that targets the transmembrane domain of Neuropilin-1 (NRP1) receptor in cancer cells. The virus conjugated nanopeptide inhibited angiogenesis and cell migration by disrupting the NRP1-PlexA1 complex and downstream [136]. In another study, Dawson and co-workers developed Cowpea mosaic virus (CPMV) NPs fused with a fluorescent PEGylated peptide and VEGFR-1 ligand for tumor targeting and imaging. These NPs showed a high affinity towards VEGFR-1 on endothelial cell lines and VEGFR1-expressing tumor xenografts in mice [137]. The vimentin overexpression in tumor endothelium shows a relationship with the CPMV uptake in tumor endothelial cells, as revealed in studies using the chick choreoallantoic membrane tumor model. The fluorescent CPMV sensors allowed for the visualization of the flow of blood and further exploited in tumor angiogenesis imaging [138]. The use of CPMV as a natural endothelial probe in imaging vascular disease may provide novel insights into the expression pattern of surface vimentin [138,139]. Similarly, a fluorescent plant VNPs with specific targeting ligands TMV-BF3 were developed for intravital imaging of the mouse brain vasculature (Figure 8). Further, these VNPs were used for delivery of therapeutic agents such as drugs or peptides and may lead to the development of novel cost-effective tools for in vivo theranostics [140].

Regardless of the enormous development in VEGF mediated antiangiogenic therapies available for therapeutic use, clinical evidence is escalating to recommend that targeting only VEGF may not be effective in inhibiting tumor angiogenesis. The epidermal growth factor-like domain 7 protein (EGFL7, 30 kDa) is only expressed by vascular remodeling endothelial cells and identified as a key controller of various angiogenic pathways [141]. Recently, researchers developed EGFL7 ligand decorated CPMV viral nanoparticles for intravital imaging of tumor neovasculature [141]. They further used ^68^Ga-labeled E7p72 radiotracer for in vivo targeting PET imaging. These studies suggest that using EGFL7 expression as a biomarker for tumor angiogenesis would be beneficial since it is expressed in cells associated with tumor blood cell remodeling and not by mature blood vessels [134,141]. Several other nanoparticles for antiangiogenic therapy were discussed in detail in the recent literature cited [13,21,22,142,143,144,145,146].

We have tabulated the recent examples of various nanomaterials used for the antiangiogenic therapy (Table 2).

## 11. Challenges of Nanomedicine, Conclusion, and Future Perspective

Various nanomaterials were comprehensively utilized for different biomedical applications including cancer theranostics and anti-angiogenic cancer therapy. Nevertheless, some of these nanomaterials occasionally show significant toxicity and other side effects causing potential challenges for successful clinical application. Hence, it is crucial to test their biosafety, long term metabolic activity, degradation property, pharmacokinetics and pharmacodynamics, interaction with immune cells, sustainable circulation in the body, etc., before their use in humans [25]. The future challenge is to develop robust cancer targeted anti-angiogenic nanomedicine with minimum side effects. Additionally, combination of FDA approved anti-angiogenic drugs to these nanomaterials can potentially increase the therapeutic effects and decrease the dose of treatment and subsequent toxicity. Additionally, vital information about NPs interaction, uptake, circulation, retention, and excretion are necessary and need to be thoroughly investigated. Apart from that, more studies are required to reduce the cost of industrial scale production, develop better technologies to synthesize multifunctional nanomaterials, and to identify the best route of drug administration [26].

The erratic structure and aberrant functionalities of tumor-associated neo-vessels provide much needed information to fight and evade the challenges associated with anticancer therapies. It is quite evident that these therapeutic modalities exhibited an efficacious management of cancer under certain settings, yet a substantial range of benefits is to be realized for many patients in the form of metastasis as well as overall survival. Moreover, such approaches and associated with untoward toxicity to normal cells along with many adverse side effects. The primary future challenge, thus, boils down to designing novel targeted nanomedicine for anti-angiogenic therapeutics. This should be accompanied by combinatorial strategy with chemotherapeutics to enhance the anticancer efficacy along with antiangiogenic tumor growth inhibition. Discovery of novel biomarkers is also urgently needed to accomplish efficacious drug treatment and their safety. Formulating new strategies to overcome drug resistance is a must as cancer cells routinely become impervious to drugs. For sure, pharmacokinetics and pharmacodynamics parameters of the nanomaterials should be assessed properly, biodegradability and clearance should be determined meticulously, and route of administration and dose regimen should be optimized accurately. Obviously, the future cancer nanomedicine will demand a more intense understanding of metabolic aspects of cancers, possibility of immune destruction, circumventing various physiological barriers, exploring material properties, etc., to achieve exceedingly efficient nanocarriers for anti-angiogenic cancer therapy.

## Figures and Tables

**Table 1:** FDA approved angiogenic inhibitors, trade name, chemical structures, target, and FDA and approved treatments.

Drug (Trade Name)	Structure	Chemical Name, Target, and FDA Approved to Treat Patients with	Ref.
Bevacizumab (Avastin^®^)	Anti-VEGF monoclonal antibody	Anti-VEGF monoclonal antibody Cervical cancer: Nonresponsive to other treatment/metastastatic/recurrent. Colorectal cancer: metastastatic. Glioblastoma: Nonresponsive to other treatment/recurrent. Nonsquamous non-small cell lung cancer: locally advanced, nonresectable/metastastatic/recurrent. Ovarian epithelial, fallopiantube, orprimary peritoneal cancer: stage III/stage IV/recurrent. Renal cell carcinoma: metastastatic.	[13,43,44,45]
Thalidomide (Synovir, Thalomid^®^)	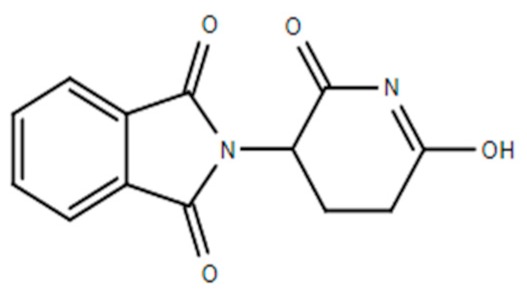	(±)-2-(2,6-Dioxo-3-piperidinyl)-1H-isoindole-1,3(2H)-dione Immune modulator and inhibits VEGF and bFGF production Multiple myeloma: newly diagnosed.	[46]
Lenalidomide (Revlimid^®^)	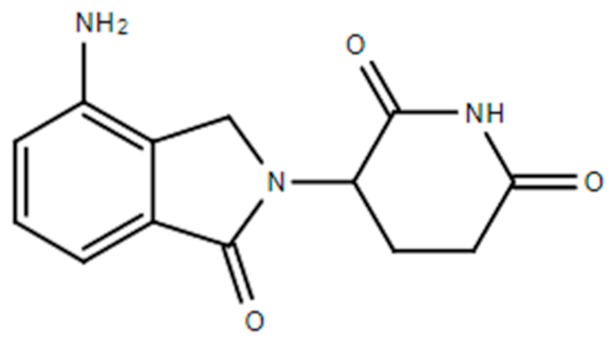	1-oxo-2-(2,6-dioxopiperidin-3-yl)-4-aminoisoindoline VEGF-induced PI3K-Akt pathway signaling and HIF-1α expression Anemia associated with certain types of myelodysplastic syndromes. Follicular lymphoma: Nonresponsive to other treatment. Mantle cell lymphoma: Nonresponsive to other treatment/recurrent. Marginal zone lymphoma: Nonresponsive to other treatment. Multiple myeloma and as maintenance therapy	[13,42,47]
Sorafenib (Nexavar^®^)	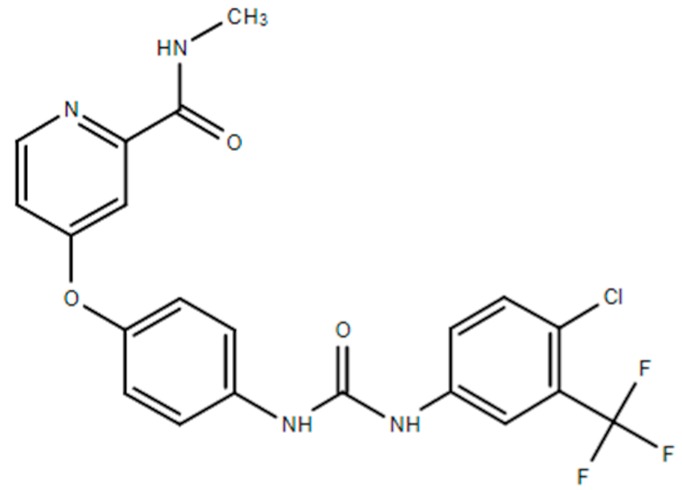	4-[4-(([4-chloro-3-(trifluoromethyl)phenyl]carbamoyl)amino)phenoxy]-N-methylpyridine-2-carboxamide Small molecule inhibitors of the VEGFR-2 tyrosine kinase activity. Hepatocellular carcinoma: Nonresectable. Renal cell carcinoma: Advanced. Thyroid cancer: Progressive/metastastatic/recurrent.	[41,48]
Sunitinib (Sutent^®^)	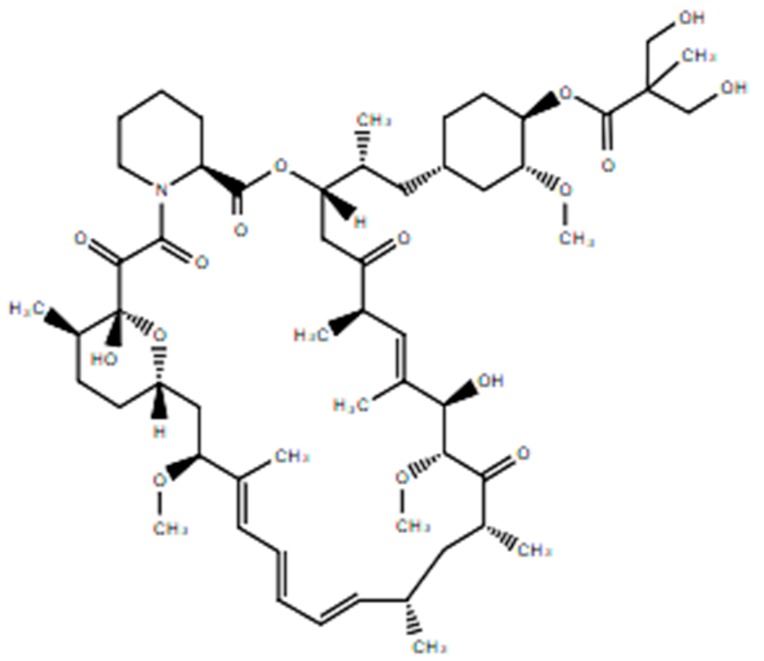	(Z)-N-(2-(diethylamino)ethyl)-5-((5-fluoro-2-oxoindolin-3-ylidene)methyl)-2,4-dimethyl-1H-pyrrole-3-carboxamide Small molecule inhibitors of the VEGFR-2 tyrosine kinase Gastrointestinal stromal tumor: nonresponsive to imatinibmesylate. Pancreatic cancer: progressive neuroendocrine tumors that are nonresectable/metastastatic. Renal cell carcinoma: advanced disease.	[13,41,42,49]
Temsirolimus	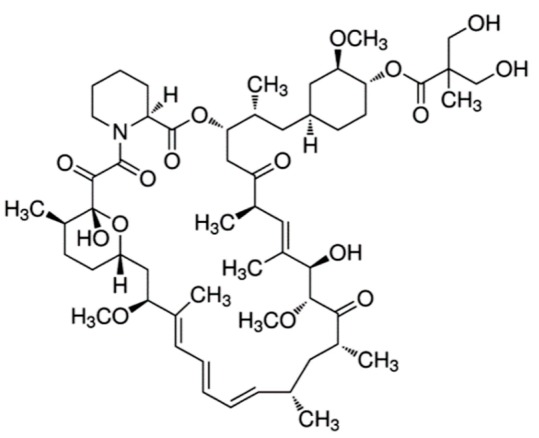	42-[3-Hydroxy-2-(hydroxymethyl)-2-methylpropanoate]-rapamycin Reduces synthesis of VEGF and targets the mammalian target of rapamycin (mTOR) Retinoblastoma. Renal cell carcinoma: advanced disease.	[50]
Axitinib (Inlyta^®^)	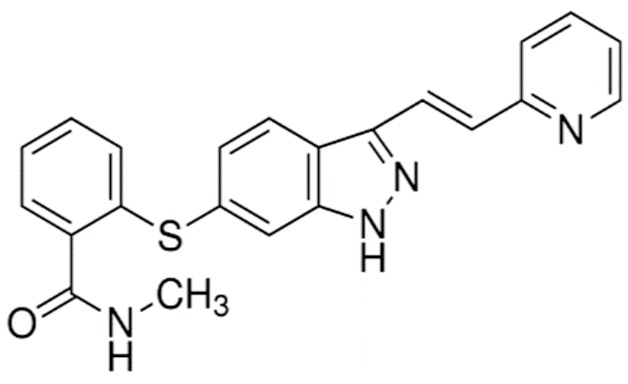	N-Methyl-2-((3-((1E)-2-(pyridin-2-yl)ethenyl)-1H-indazol-6-yl)sulfanyl)benzamide Inhibitor of VEGF-1, -2, and -3 Renal cell carcinoma: Advanced/nonresponsive to other treatment.	[13,42,51]
Pazopanib (Votrient^®^)	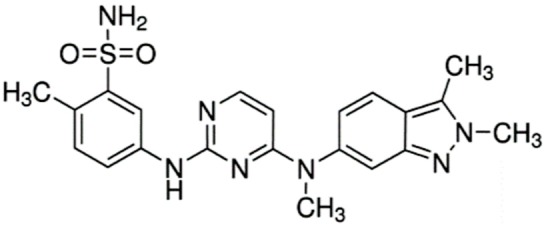	5-((4-((2,3-dimethyl-2H-indazol-6-yl)(methyl)amino)pyrimidin-2-yl)amino)-2-methylbenzenesulfonamide Small molecule multi-targeted receptor tyrosine kinase inhibitor Renal cell carcinoma: Advanced. Soft tissue sarcoma: Advanced. Nonresponsive to other treatment.	[13,52]
Lenvatinibmesylate (Lenvima^®^)	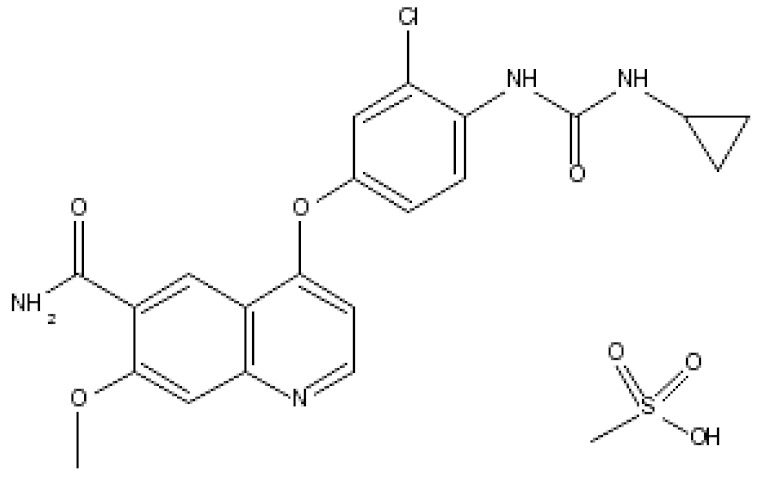	4-(3-chloro-4-(3-cyclopropylureido)phenoxy)-7-methoxyquinoline-6-carboxamide methane sulfonate Lenvatinib inhibits tyrosine kinase activity of VEGF1, 2 and 3, fibroblast growth factor receptors (FGFRs) 1–4 Endometrial carcinoma: Advanced/nonresponsive to other treatment. Hepatocellular carcinoma: first-line treatment in nonresectable tumor. Renal cell carcinoma: Advanced. Thyroid cancer: Progressive/recurrent/metastatic/nonresponsive to radioactive iodine treatment.	[53]
Cabozantinib (Cometriq^®^)	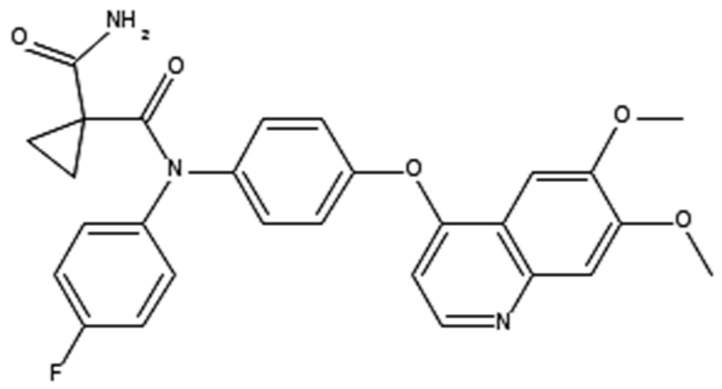	1,1-cyclopropanedicarboxamide, n′-[4-[(6,7-dimethoxy-4-quinolinyl)oxy]phenyl]-n-(4-fluorophenyl)- c-MET and VEGFR2 Inhibitor Hepatocellular carcinoma: already been treated with sorafenib. Medullary thyroid cancer: Progressive/metastatic. Renal cell carcinoma: Advanced.	[41,54]
Everolimus (Afinitor^®^)	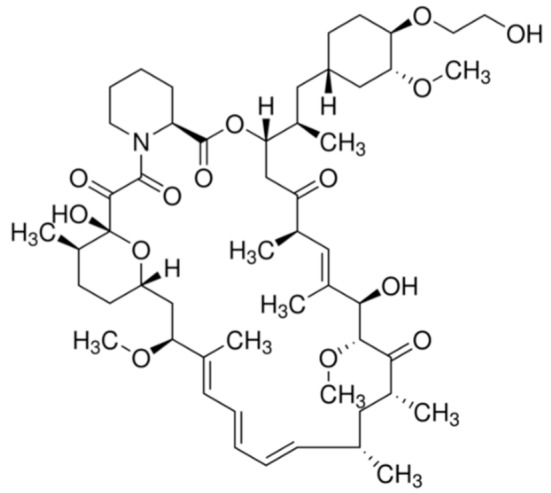	(1R,9S,12S,15R,16E,18R,19R,21R,23S,24E,26E,28E,30S,35R)-1,18-dihydroxy-12-((2R)-1-[(1S,3R,4R)-4-(2-hydroxyethoxy)-3-methoxycyclohexyl]propan-2-yl)-19,30-dimethoxy-15,17,21,23,29,35-hexamethyl-11,36-dioxa-4-azatricyclo[3 0.3.1.0(4,9)]hexatriaconta-16,24,26,28-tetraene-2,3,10,14,20-pentone40-O-(2-hydroxyethyl)-rapamycin Immunosuppression and targets the mTOR pathway Breast cancer: Advanced hormone receptor–positive (HR+) breast cancer that is also HER2 negative. Pancreatic cancer, gastrointestinal cancer, and lung cancer: Neuroendocrine tumors/nonresectable/metastatic. Renal cell carcinoma: Advanced. Subependymal giant cell astrocytoma: Nonresectable.	[13,41,55]
Vandetanib (Caprelsa^®^)	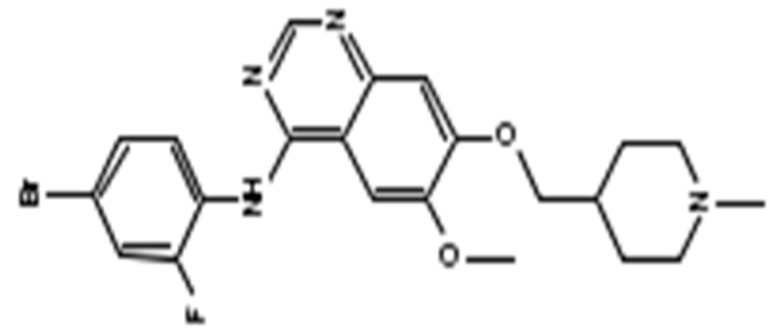	(4-Bromo-2-fluoro-phenyl)-[6-methoxy-7-(1-methyl-piperidin-4-ylmethoxy)-quinazolin-4-yl]-amine Dual Inhibitor of VEGFR and Epidermal Growth Factor Receptor (EGFR) Tyrosine Kinases and also inhibits the mTOR–HIF-1 alpha–VEGF signaling axis Medullary thyroid cancer: Nonresectable/metastatic.	[13,41,42,56]
Ramucirumab (Cyramza^®^)	Anti-VEGFR2 monoclonal antibody	Anti-VEGFR2 monoclonal antibody Colorectal cancer: Metastatic/nonresponsive to other treatment like bevacizumab, oxaliplatin, and fluoropyrimidine. Hepatocellular carcinoma: Nonresponsive to sorafenib. Non-small cell lung cancer: Metastatic/aggravated after platinum chemotherapy/with a mutation in the EGFR gene or ALK gene. Stomach adenocarcinoma or gastroesophageal junction adenocarcinoma: Advanced/metastatic	[41,57]
Regorafenib (Stivarga^®^)	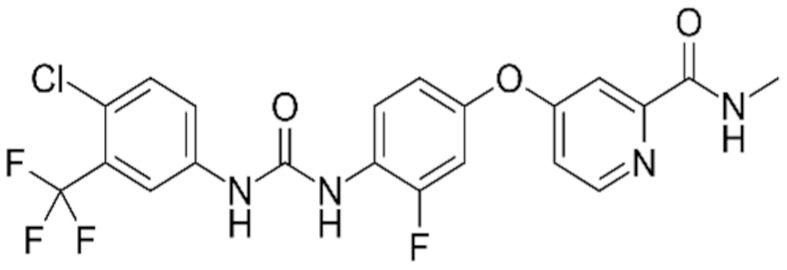	4-(4-(3-(4-Chloro-3-(trifluoromethyl)phenyl)ureido)-3-fluorophenoxy)-N-methylpicolinamide. Dual targeted VEGFR2-TIE2 tyrosine kinase inhibition. Colorectal cancer: Metastatic/nonresponsive to other treatment. Gastrointestinal stromal tumor: Advanced/nonresectable/metastatic/nonresponsive to imatinibmesylate and sunitinib malate. Hepatocellular carcinoma: Nonresponsive to sorafenib.	[41,58]
Ziv-aflibercept (Zaltrap^®^)	A recombinant fusion protein comprising the extracellular domains of human VEGF receptors 1 and 2	Inhibitor of VEGF Colorectal cancer: Metastatic/nonresponsive to other treatment.	[41,59]

**Table 2:** Various nanomaterials and their anti-angiogenesis applications.

S.No	Nanoparticle	Therapeutics	Application	Ref
1	Liposomes	Honokiol (potent anti-angiogenesis agent)	Liposomal honokiol improved efficacy of radiotherapy and chemotherapy in lung andovarian tumors.	[147,148,149]
2	Liposomes	Gd-DTPA Rhodamine PE	Gd-RGD-liposomes for target-specific MRI imaging and therapy of tumor angiogenesis.	[150]
3	Liposomes	Anginex-peptide	Anginex-liposomes used imaging for the angiogenesis-dependent disease.	[151]
4	Liposomes	EverolimusmTOR) EG00229 (VEGF/NRP1)	Showed effective tumor growth inhibition in a highly aggressive syngeneic immune-competent mouse model.	[152]
5	Solid-lipid nanoparticle	Bevacizumab	BSLNPs showed highly more effective than the parent in glioblastoma.	[153]
6	Liposomes	Fenretinide	Fenretinide–liposomes showed enhanced antiangiogenic and antitumor activity on human neuroblastoma.	[154]
7	Liposomes	ALK-siRNA	ALKsiRNA loaded liposomes induce apoptosis and inhibit angiogenesis.	[155]
8	Liposomes	Clodronate	Clo-liposomes efficiently deplete tumor-associated macrophages and showed antiangiogenic and antitumor effects in primary and metastatic melanoma.	[156]
9	Gold nanoparticles	Recombinant human endostatin (antiangiogenic agent)	Endostatin-gold nanoparticles normalized vessels in metastatic colorectal cancer.	[116]
10	Gold nanoparticles	GNPs	Gold nanoparticles inhibit subsequent angiogenesis-related signaling events.	[67]
11	Gold nanoparticles	Quercetin	Quercetin-GNPs inhibits EMT, angiogenesis and invasiveness in cancer.	[118,157,158,159,160]
12	Gold NPs	Peptides	Inhibit angiogenesis.	[161,162,163]
13	Nanoparticles	Small molecules	Inhibits tumor angiogenesis and tumor growth.	[99,142,143,164,165]
14	Lipid conjugates	PTX/LGC	IRGD-nanoconjugates improve tumor vessel normalization to achieve optimal chemo drug delivery into solid tumors.	[144]
15	PLA -NPs	Delta-like ligand 4 (Dll4-GD16-PTX	GD16-PTX-NPdemonstrated significant antiangiogenic and anticancer activity.	[166]
16	Cerium oxide-NPs	Nanoceria (NCe)	NCe-FA demonstrated excellent antiangiogenic effect in ovarian cancer.	[167,168]
17	Tetrac-NP	Tetraiodothyroacetic acid	Tetrac-NP significantly suppressed tumor growth and angiogenesis in murine xenograft models.	[169]
18	Polymeric Nanoparticle	Diamino Propane Tetraiodothyroacetic Acid	NPs showed excellent pharmacokinetics, biodistribution, and antiangiogenesis properties.	[170]
19	Carbon-NPs	Angiogenesis inhibitors	Inhibits tumor angiogenesis and tumor growth.	[146,171]
20	Silver nanoparticles (Ag-NPs)	Ag-NPs	Ag-NPs inhibit vascular endothelial growth factor (VEGF) and the formation of new blood microvessels.	[120,172]
21	Chitosan nanoparticles (CNP)	Alphastatin/CNPs	AsCs-NPs inhibited the SphK1-S1P signaling pathway and enhanced the antiangiogenic effect of Alphastatin both in vitro and in vivo.	[173,174]
22	Graphene-NPs	Graphite(G), rGOandnGO	Graphite nanoparticles and graphene oxide nanoplatelets showed potential antiangiogenic effects.	[175,176]
23	Chitosan-derived micelles	Apatinib	Apatinib-micelles showed effective anti-angiogenesis cancer therapy.	[177]
24	Cationic PEGylated liposomes	Gambogic acid	GAL significantly inhibited angiogenesis against TNBC.	[178]
25	PLGA copolymer	Osseltamivirphosphate (OP)	PLGA-OP actively impedes tumor neovascularization, growth, and metastasis in a mouse model of human pancreatic carcinoma.	[179]
26	Lipid-PA nanoparticles	Rapamycin and DiR	RDLPNPs showed an excellent antitumor effect with the enhanced photothermal and antiangiogenic effect.	[180]
27	Selenium nanoparticles	VEGF siRNA	Showed enhanced in vivo VEGF-siRNA silencing and fluorescence imaging efficacy.	[113]
28	Mesoporous silica nanoparticle	Combretastatin A4 doxorubicin	Tumor vascular-targeted co-delivery iRGD-NPs presented excellent anti-angiogenesis and antitumor activity.	[181]
29	pH-sensitive polymeric nanoparticles	Doxorubicin curcumin	Displaced enhanced proapoptotic and antiangiogenic activities.	[182]
30	Multifunctional nanodrugs	LMWH and ursolic acid	Demonstrated excellent anti-angiogenesis and antitumor activity.	[183]
